# A case report of long-latency evoked diaphragm potentials after exposure to acute intermittent hypoxia in post-West Nile virus meningoencephalitis

**DOI:** 10.1152/jn.00406.2024

**Published:** 2024-12-30

**Authors:** Joseph F. Welch, Erica A. Dale, Jayakrishnan Nair, Paul W. Davenport, Emily J. Fox, Gordon S. Mitchell

**Affiliations:** 1 School of Sport, Exercise and Rehabilitation Sciences, College of Life and Environmental Sciences, University of Birmingham, Birmingham, United Kingdom; 2 Breathing Research and Therapeutics Center, Department of Physical Therapy, University of Florida, Gainesville, Florida, United States; 3 McKnight Brain Institute, University of Florida, Gainesville, Florida, United States; 4 Department of Physiology and Aging, University of Florida, Gainesville, Florida, United States; 5 Department of Physiological Sciences, University of Florida, Gainesville, Florida, United States; 6 Department of Physical Therapy, Thomas Jefferson University, Philadelphia, Pennsylvania, United States; 7 Brooks Rehabilitation, Jacksonville, Florida, United States

**Keywords:** diaphragm, neuroinflammation, phrenic, plasticity, West Nile virus

## Abstract

We present a case report of a 42-year-old female with post-West Nile virus meningoencephalitis who exhibited unique, long-latency diaphragm potentials evoked by transcranial and cervical magnetic stimulation after exposure to acute intermittent hypoxia (AIH). The subject was recruited for a study investigating AIH effects on respiratory motor function in healthy individuals. She had contracted West Nile virus infection 5 years before assessment that resulted in hospitalization and persistent allodynia but was not reported to the research team. During the study, transcranial (TMS) and cervical (CMS) magnetic stimulation were performed before and 30–60 min after a single presentation of AIH [15, 1-min hypoxic episodes (~9% inspired O_2_), with 1-min normoxic intervals]. Diaphragm EMG was recorded using chest wall surface electrodes. At baseline, evoked diaphragm potentials were within normal ranges for both TMS (onset latency = 17.0 ± 1.1 ms; peak-to-peak amplitude = 220 ± 27 μV) and CMS (onset latency = 7.8 ± 0.6 ms; peak-to-peak amplitude = 336 ± 8 μV). However, long-latency TMS- and CMS-evoked potentials were observed 30–60 min post-AIH that were not present at baseline nor in healthy subjects. The onset of long-latency potentials ranged from 50 to 808 ms. While AIH is a potentially useful therapeutic strategy to enhance motor function after neurological disease or injury, it may elicit distinct effects in individuals with a history of neuroinfectious disease. Possible explanations for these unusual responses are discussed.

## NEW & NOTEWORTHY

A 42-year-old female with post-West Nile virus meningoencephalitis demonstrated long-latency diaphragmatic potentials evoked by transcranial and cervical magnetic stimulation following exposure to acute intermittent hypoxia that were not present at baseline nor in healthy subjects. Although the cause of long-latency responses is unknown, we discuss possible mechanisms whereby acute intermittent hypoxia could create unique effects on the diaphragm/phrenic motor system in individuals with a history of neuroinfectious disease.

## Graphical Abstract

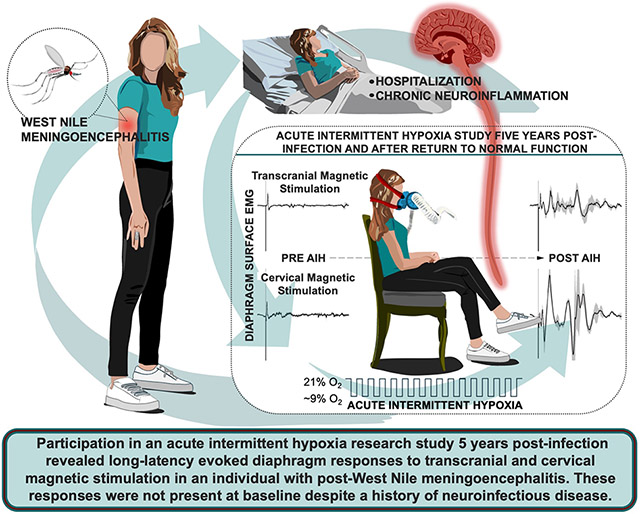

## INTRODUCTION

Acuteintermittent hypoxia (AIH) is a novel strategy to strengthen synaptic inputs to respiratory motor neurons and enhance respiratory motor output (1, 2). Although cellular mechanisms giving rise to AIH-induced respiratory motor plasticity are well described in the rodent phrenic motor system (3, 4), little work has been done concerning mechanisms of AIH-induced plasticity in humans. We recently conducted a study of diaphragm potentials evoked by transcranial (TMS) and cervical magnetic stimulation (CMS) to assess central versus peripheral adaptations in the phrenic/diaphragm motor system after AIH (5). In our prior experiment, healthy individuals with no known history of neurological impairment were studied; data were also collected from one individual with an undisclosed history of West Nile virus (WNV) meningoencephalitis. While data from this subject were not included in published work, the results were unique and potentially important as we seek to understand AIH effects in diverse populations. Here, we present those data as a case report describing long-latency evoked diaphragm potentials revealed by AIH exposure.

## MATERIALS AND METHODS

### Case Presentation

First detected in the United States in 1999, WNV infects <5% of people in affected populations (6). From 1999 to 2019, 501,801 confirmed cases were reported to the Centers for Disease Control and Prevention in the United States (7). Of these cases, <1% of individuals developed central nervous system infection (encephalitis). The prognosis for WNV encephalitis is poor; the mortality rate is ~10% for those hospitalized (8). In the acute phase, neck stiffness, disorientation, respiratory distress, tremors, paralysis, and seizures are common and may become permanent (9). In rodents, WNV infection suppresses diaphragm electrical activity (10) and impairs phrenic nerve activity elicited by optogenetic activation of phrenic motor neurons (11). Thus the main cause of respiratory deficits following WNV infection appears to be due to lower motor neuron pathology versus impaired respiratory drive (12).

The following account of WNV symptoms, medical evaluation, and treatment was provided by the subject, who has professional knowledge of neurologic conditions and kept detailed records of her medical care. Five years before participation in the study, the subject developed flu-like symptoms that progressively worsened over 1 wk. Upon admittance to urgent care, blood pressure and body temperature were recorded, and radiographs were taken. The subject was severely dehydrated with possible lung infection/inflammation. She was given intravenous fluids and an anti-emetic injection and discharged with a tentative diagnosis of pneumonia. Symptoms worsened over the next 2 days (e.g., inability to walk without assistance). Upon hospital readmittance, urinalysis and blood tests were uninformative; thus cerebrospinal fluid was sampled via the lumbar spine. Polymerase chain reaction analyses of the cerebrospinal fluid led to a diagnosis of WNV meningoencephalitis, which was reported to the Centers for Disease Control and Prevention. The cause of WNV infection was unknown, although an outbreak of this mosquito-borne disease was reported in the area.

The subject remained in an isolated hospital suite and was treated with intravenous fluids, opioid analgesics, and an intermittent pneumatic compression device placed around the lower body to improve circulation. Spirometry and blood tests were normal, and she was discharged 1 wk later. Over the next 5 mo, the subject was bedridden, developed muscle atrophy, and was only able to ambulate to the bathroom. An infectious disease neurologist noted that the subject had significant loss of somatosensation of her abdomen and thighs, accompanied by deficits in proprioception and spasticity of the lower limbs. She was prescribed baclofen, clonazepam, gabapentin, and tizanidine (in succession), in addition to magnesium and a vitamin D and B complex. The subject experienced deep muscle and joint pain and allodynia described as a sunburn sensation; she was unable to raise her arms above her head and perform fine motor movements, and she developed occasional opsoclonus, as has been reported in other cases (13, 14). She partook in 10 mo of physical rehabilitation, primarily focused on regaining a normal gait pattern. At the time of the study visit, physical function had returned; the subject was physically active and experienced occasional “flare-ups” of neuropathic pain, usually induced by exercise. Cutaneous allodynia and proprioceptive deficits had not subsided, despite recovered muscle mass/strength.

### Screening, Enrollment, and Informed Consent

The subject was initially contacted by study investigators to gauge interest in participating in an experiment examining the effect of AIH on cortico-diaphragmatic conduction in healthy humans, approved by the University of Florida Institutional Review Board (IRB2019001170). Before the visit, the subject completed a prescreening health questionnaire that asked about her history of cardiovascular, respiratory, and neuromuscular disease/injury. The subject did not disclose any information that constituted exclusion from the study. Upon arrival at the laboratory, height (178 cm), weight (68 kg), resting blood pressure (121/78 mmHg), and peripheral capillary oxyhemoglobin saturation (SpO_2_; 98%) were measured. The subject was enrolled after providing written informed consent.

Once the study was completed, the subject was contacted by study investigators due to her unusual responses to the experimental protocol. During these discussions, the subject informed study investigators of her previous WNV diagnosis, which was then reported to the Institutional Review Board. Data were subsequently removed from the approved study data set, and permission was granted to use them in a case report with subject consent (available upon request).

### Magnetic Stimulation and Electromyography

Please refer to Welch et al. (5) for detailed methodological information. In brief, transcranial (TMS) and cervical (CMS) magnetic stimulation were performed to induce diaphragmatic motor-evoked potentials (MEP) and compound muscle action potentials (CMAP), respectively, according to established standards (15, 16). Two blocks of stimuli were delivered before AIH (for reliability purposes), separated by 30 min. A third block of stimuli was delivered 30–60 min after AIH to compare changes in MEP and CMAP amplitudes pre- versus post-AIH.

For TMS, the subject was seated comfortably. The vertex of the skull was identified by the intersection between nasion to inion and tragus to tragus. Stimuli were delivered using a handheld 70-mm figure-of-eight remote coil (3190-00; Magstim, Whitland, Wales, UK) powered by a magnetic stimulator (200-2; Magstim). The coil was held over the vertex with current flowing in the anteroposterior direction. The coil was moved around the vertex and rotated in 45° installments until the largest MEP was observed. This location was marked on the scalp to ensure accurate coil positioning in future stimulations. Recruitment curves were plotted by gradually increasing the stimulus intensity from 60 to 100% of the maximal stimulator output in 5% increments in random order. A minimum of seven stimuli were delivered at each intensity separated by 20–30 s. Stimuli were delivered at end expiration, as determined using a pie-zoelectric respiration transducer (1132, Pneumotrace II; UFI, Morro Bay, CA) placed securely around the abdomen.

For CMS, the subject was seated comfortably with the neck flexed. A 90-mm handheld circular coil (9784-00; Magstim) was placed over the cervical spine between C3 and C7, with the current flowing clockwise. The optimal stimulation site was identified by gradually moving the coil until the largest CMAP was observed. Stimulus-response curves were plotted as per TMS, with the lowest stimulation intensity delivered at 40% of the maximal stimulator output. A minimum of three stimuli were delivered at each intensity separated by 20–30 s. Stimuli were delivered at end expiration.

Surface Ag/AgCl electrodes (6801; The Prometheus Group, Dover, NH) were placed over the seventh intercostal space along the anterior axillary line to record right costal diaphragm EMG activity. The ground electrode was placed on the acromion process of the scapula. Skin was lightly abraded and cleaned before electrode placement.

### Acute Intermittent Hypoxia

Hypoxia (~9% inspired O_2_) was delivered via a facemask (7900; Hans Rudolph, Kansas City, MO) attached to a hypoxia generator (GO2altitude; Biomedtech, Melbourne, Australia). The AIH protocol consisted of 15, 1-min hypoxic episodes interspersed with 1-min intervals breathing ambient air (~21% inspired O_2_). Cardiovascular responses (heart rate, blood pressure, and SpO_2_) were monitored throughout and recorded once every 3 AIH cycles.

### Data Processing and Analyses

Evoked diaphragm potentials were analyzed using a previously detailed procedure (15, 16) for onset latency (difference in time between stimulus artifact and MEP/CMAP onset), duration (difference in time between MEP/CMAP onset and off-set), peak-to-peak amplitude (voltage difference between positive and negative peaks), and total rectified area (area of full wave rectified EMG between MEP/CMAP onset and off-set). Diaphragm EMG (EMG_di_) burst frequency and amplitude were assessed during eupnea before and 30 min after AIH (immediately before magnetic stimulation) using the same technique as previously described (5). Signals were amplified and band-pass filtered (0.01–1 kHz; ML-132; ADInstruments, Colorado Springs, CO), sampled at 10 kHz (PowerLab 8/35; ADInstruments), and monitored online using LabChart data acquisition software (V8.1; ADInstruments).

## RESULTS

The subject completed the full study protocol without difficulty or discomfort. However, ~1 day after the experiment, the subject experienced muscle and joint soreness in her neck and shoulders, as well as a self-described “flare-up” of generalized body aches and of “feeling hot,” as if febrile. Self-measured oral temperature readings were normal.

### Evoked Diaphragm Potentials

**Figure 1.**
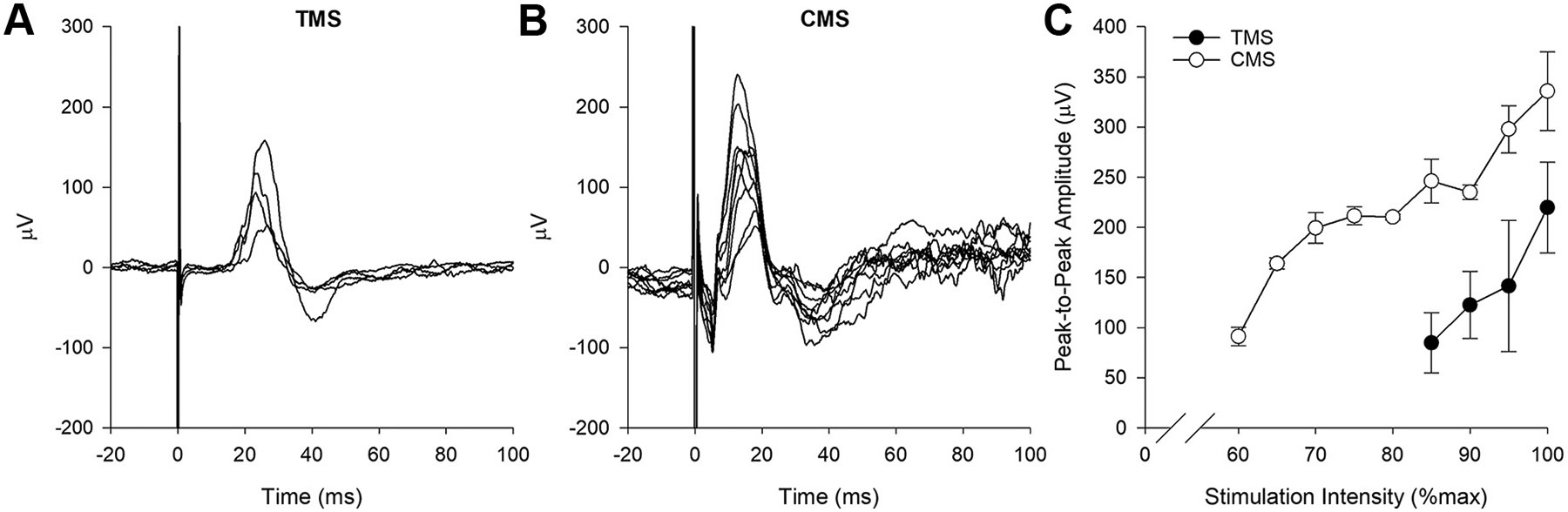
Stimulus response curves. Example diaphragm potentials evoked by transcranial (TMS; *A*) and cervical (CMS; *B*) magnetic stimulation of gradually increasing intensity. Recruitment thresholds for TMS- and CMS-evoked responses were 85% and 60% of the maximal stimulator output, respectively (*C*).

Stimulus-response curves are shown in Fig. 1. At baseline, maximal MEP and CMAP peak-to-peak amplitudes were on average, 220 ± 27 μV and 336 ± 8 μV, respectively. Within-day between-block coefficients of variation for MEP and CMAP amplitudes were 38.8% and 10.6%, respectively (Table 1). Onset latencies for TMS- and CMS-evoked potentials were 17.0 ± 1.1 and 7.8 ± 0.6 ms, respectively.

**Figure 2.**
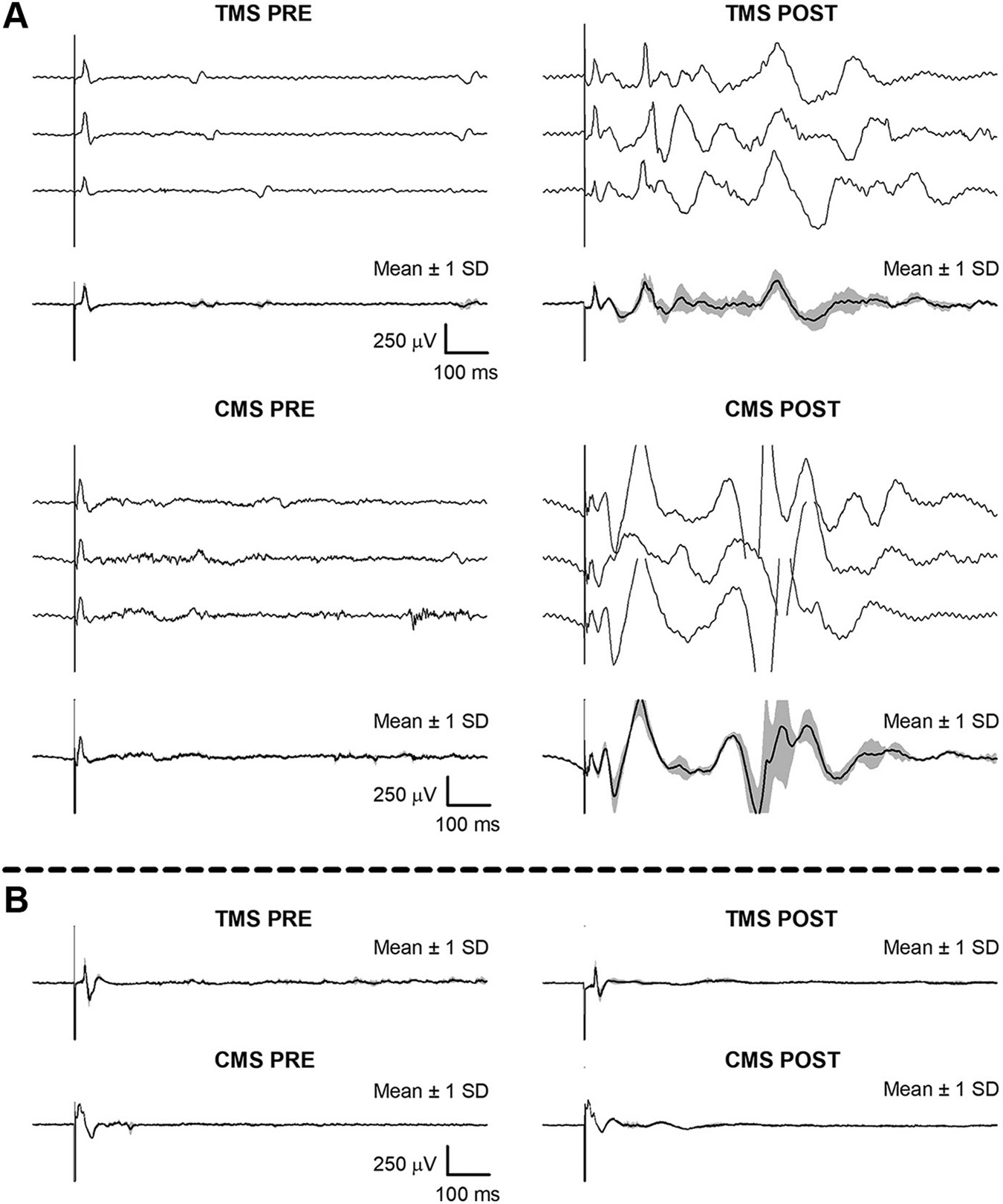
Evoked diaphragm responses to transcranial (TMS) and cervical (CMS) magnetic stimulation before (PRE) and 30–60 minutes after (POST) exposure to acute intermittent hypoxia. *A*: example data recorded from the female subject with post-West Nile virus meningoencephalitis. *B*: example data recorded from a representative healthy participant from the associated published work (5). Thick black line represents the average of all stimulations at a given time point. Shaded gray area indicates ± 1 SD around the mean.

Normal evoked responses were present at baseline (Fig. 2, A and B, *left*) as compared to established normative values (15, 17). However, following AIH, long-latency peaks appeared after every cortical and cervical stimulation (Fig. 2, A and B, *right*). For TMS, four long-latency peaks were detected on average (peak-to-peak amplitude ≥50 μV; minimum = 3 peaks; maximum = 8 peaks) at latencies ranging from 146 to 808 ms. For CMS, six long-latency peaks were detected on average (minimum = 4 peaks, maximum = 9 peaks) at latencies ranging from 50 to 745 ms. Long-latency evoked diaphragm potentials were not present at baseline nor were they observed in healthy individuals (5).

### Diaphragm Activation

**Figure 3.**
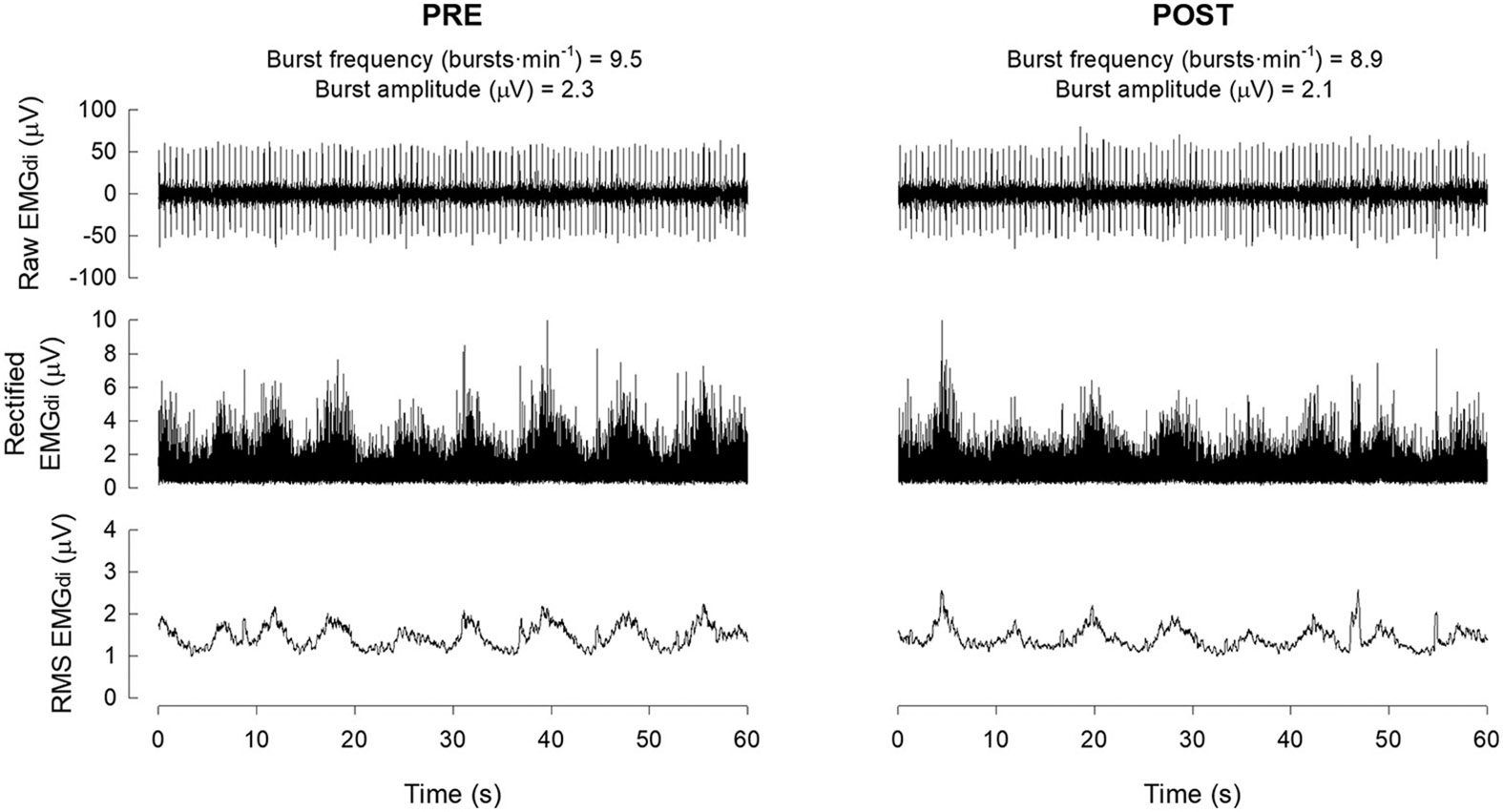
Diaphragm EMG activity before (PRE) and 30–60 minutes after (POST) acute intermittent hypoxia. Diaphragm EMG (EMG_di_) activity was recorded during eupnea PRE and POST using surface electrodes placed on the chest wall. RMS, root mean square. See text for more information.

Eupneic EMG_di_ burst frequency decreased marginally from 9.5 bursts·min^−1^ pre-AIH to 8.9 bursts·min^−1^ post-AIH. Burst amplitudes also decreased marginally from 2.3 μV pre-AIH to 2.1 μV. Example traces are shown in Fig. 3. Baseline EMG_di_ burst frequencies and amplitudes were lower than healthy individuals (12.1 ± 3.8 bursts·min^−1^ and 5.9 ± 1.8 μV, respectively) as reported in our previous study (5).

### Cardiorespiratory Responses to Acute Intermittent Hypoxia

On average, SpO_2_ fell to 85% during hypoxic episodes and returned to within 4% of baseline during normoxic intervals. Heart rate ranged from 89 beats·min^−1^ at baseline to a peak of 96 beats·min^−1^ during hypoxic episodes. Mean arterial pressure was 92 mmHg at baseline and varied between 90 and 99 mmHg during hypoxic episodes (Table 2). These responses were similar to healthy individuals (5).

## DISCUSSION

### Main Findings

In this case report, an adult female with post-WNV meningoencephalitis demonstrated long-latency TMS- and CMS-evoked potentials in the diaphragmatic surface EMG after exposure to AIH that were not observed at baseline and are not observed in healthy individuals who do not have any known history of WNV or other neuroinfectious diseases (5, 16, 18). The mechanism of these long-latency responses is unknown. However, we speculate that long-latency potentials reflect novel forms of AIH-induced plasticity caused by persistent changes in the central nervous system due to prior WNV infection.

### Long-Latency Evoked Diaphragm Potentials

Many possibilities exist concerning the origin of long-latency peaks in the diaphragm EMG after AIH exposure in this case of post-WNV meningoencephalitis. Below, we briefly outline three potential mechanisms that may explain these unusual responses.

#### Activation of thin-fiber phrenic afferents.

Demonstration of pupil dilation after application of acid or alkali, electrical or thermal stimuli, or pinching of the diaphragm provided the first empirical evidence of sensory afferent pathways via the phrenic nerve (19). Small-diameter thinly myelinated Aδ fibers (group III) and unmyelinated C fibers (group IV) respond to mechanical, thermal, or chemical stimuli. Collectively, group III/IV fibers comprise the majority (~75%) of phrenic afferents (20).

Upon diaphragm contraction, group III/IV fibers may be activated, as with other skeletal muscles (21). Thin-fiber phrenic afferents enter the spinal dorsal horn where they synapse onto second order neurons that project to multiple spinal and supraspinal regions, including the somatosensory cortex (22), medulla (nucleus tractus solitarius and ventral respiratory group) (23), and spinal interneurons forming connections with thoracic intercostal α-motor neurons (24). It is not possible to differentiate between the specific fiber types that may have been activated by TMS or CMS nor can we delineate the central neural integration of those pathways. Nevertheless, we can estimate the expected latencies of responses from different neural pathways that may have been activated based on known distances and conduction velocities.

The length of the phrenic nerve is ~250–400 mm in human adults (25, 26). Since phrenic motor neurons conduct at 48–78 m·s^−1^ (27, 28), phrenic nerve conduction time is estimated to be between 3 and 8 ms (15). On the other hand, Aδ and C fibers conduct much more slowly (2.6–30 and 0.5–2.5 m·s^−1^, respectively), with estimated conduction times from 8 to 153 ms for Aδ fibers and 100 to 800 ms for C fibers (29). Cortical (“upper”) motor neurons projecting to the cervical spinal cord are ~270 mm in length (30); thus central motor conduction times (from motor cortex to cervical spinal cord) are estimated to be between 8 and 12 ms (16, 17). Based on the above information, after diaphragm contraction (elicited by the magnetic pulse), the time for Aδ- or C-fiber impulses to reach the cervical spinal dorsal horn, relay to the somatosensory cortex via a long-loop transcortical reflex (31), and return to cervical spinal phrenic motor neurons is estimated to be up to 825 ms. This estimate compares favorably with long-latency TMS- and CMS-evoked responses observed in the present case report of post-WNV infection after exposure to AIH (50–808 ms). Thus it is possible that WNV-sensitized diaphragm/phrenic afferent neurons and/or their central neural integration to produce long-latency evoked responses after AIH exposure. While there are reports of somatosensory deficits in humans with WNV (32, 33), there are no clear studies of phrenic afferent activity post-WNV, per se.

#### Enhanced excitability of central neural circuits.

In spinal C1-C2 acutely hemisected rats, systemic injection of the serotonin precursor 5-hydroxytryptophan (5-HTP) revealed long-latency contralateral phrenic nerve responses to electrical stimulation of the C2 dorsolateral funiculus. These long-latency responses were abolished by the broad-spectrum serotonin receptor antagonist methysergide and were not observed in rats that had not received 5-HTP (34). Since the same effect occurs with direct intracellular current injections into phrenic motor neurons (35), the serotonin effects reported in these studies likely arise from their ability to depolarize phrenic motor neurons, uncovering otherwise ineffective (“silent”) spinal synaptic pathways. Thus serotonin (and other neurochemicals) indirectly reveal previously silent, long-latency central neural pathways to phrenic motor neurons. Spinal synaptic pathways are also enhanced in rats given intrathecal adenosine 2_A_ receptor agonist injections (36) or exposed to AIH for 7 consecutive days (37).

Brainstem raphe neuron activation triggers serotonin release throughout the neuraxis, triggering AIH-induced phrenic motor plasticity via activation of G_q_-coupled 5-HT_2_ receptors (38-40). Thus AIH enhances excitatory connections between cervical spinal interneurons while diminishing inhibitory connectivity, thereby contributing to increased phrenic motor output (41), possibly via modification of the cervical propriospinal network by serotonin, as explained above (42). Following WNV encephalitis, AIH-induced activation of the raphe serotonergic neural network could activate dormant respiratory premotor neurons or enhance propriospinal connectivity to phrenic motor neurons. Such local (spinal) effects could be expressed in both the MEP and CMAP in this individual with a history of WNV encephalitis.

#### Adenosine-mediated activation of central and/or peripheral neural circuits.

West Nile virus encephalitis causes neuroinflammation and long-term neuromuscular deficits (43, 44). Glial cell activation is believed to be a key pathogenic factor (45). Both mild inflammation (46) and hypoxia evoke spinal adenosine accumulation (47), and adenosine can drive phrenic motor plasticity via G_s_-coupled adenosine 2_A_ receptor activation (46, 48). It may be that, in individuals with a history of neuroinflammation (e.g., post-WNV), “moderate” AIH constitutes a “severe” dose of hypoxia and adenosine accumulation that could enhance or reveal silent spinal synaptic pathways to phrenic motor neurons, similar to that described above for serotonin. For example, acute cervical spinal adenosine 2_A_ receptor agonist injections elicit phrenic motor facilitation and reveal longer latency synaptic inputs to phrenic motor neurons in rats (36). Since neuroinflammation increases spinal cord adenosine levels, WNV could, through persistent effects on spinal microglia and adenosine formation, reveal long-latency evoked responses in the phrenic/diaphragm motor system (36, 47).

Alternatively, glial ATP release could activate P2X4 receptors on “primed” microglia, triggering new synthesis and release of brain-derived neurotrophic factor in the spinal dorsal horn (49). Subsequent tyrosine kinase B receptor activation downregulates the potassium-chloride cotransporter KCC2, raising intracellular chloride levels, undermining chloride-dependent synaptic inhibition, and increasing neuronal excitability (50). Such an effect could enhance sensory afferent transmission in the spinal dorsal horn, revealing previously silent phrenic afferent connections to phrenic motor neurons. As a result, long-latency evoked responses to TMS and CMS may appear in the diaphragm EMG.

### Considerations

To our knowledge, this is the first report of TMS- and CMS-evoked diaphragmatic potentials with similarly long latencies. Although we cannot definitively link our findings with this subject’s prior diagnosis of WNV meningoencephalitis, it is the lone variable that appears to explain our novel results. Further, given the impact of neuroinflammation, or a history of neuroinflammation on AIH-induced phrenic motor plasticity (51-54), it is possible that individuals with a history of postviral syndromes, such as SARS-CoV-2, could also exhibit long-latency-evoked responses after exposure to AIH. Further work is needed to understand if these unusual responses occur in others with similar post-viral infections and/or neuroinflammation. The functional consequences of long-latency responses, as well as their underlying cause, remain to be determined.

In this case report of post-WNV, we found suppressed baseline (pre-AIH) EMG_di_ activity (burst frequency and amplitude) relative to healthy individuals (5). These findings are consistent with previous reports in hamsters, hypothesized to be caused by altered vagal afferent function (10). Future work should determine, in a larger cohort of individuals, if EMG_di_ suppression is a common feature of postviral (or septic) infections, including WNV and other forms of encephalitis.

### Conclusions

We observed unique long-latency TMS- and CMS-evoked potentials in the diaphragm EMG after AIH exposure in an individual with post-WNV infection and meningoencephalitis. While the cause of long-latency potentials is unknown, we put forward three possible explanations. First, onset latencies of evoked responses are congruent with estimated conduction times involving activation of thin-fiber phrenic afferents and spinal or supraspinal reflex pathways. Second, AIH-induced serotonin release may reveal previously silent synaptic pathways to phrenic motor neurons and/or enhance functional connectivity to cervical spinal interneurons that underlie long-latency diaphragm evoked potentials. Third, AIH-induced adenosine release from microglia may be augmented by a history of WNV infection, strengthening previously silent spinal synaptic pathways to phrenic motor neurons and revealing long-latency synaptic pathways in spinal or supraspinal circuits. Indeed, all three mechanisms (and others not considered here, such as muscle fasciculation/hyperreflexia) could contribute to long-latency evoked responses following AIH in this individual. These data may have important implications since AIH is being investigated as a possible therapeutic modality to improve motor functions in people with impaired movement (e.g., spinal cord injury, amyotrophic lateral sclerosis, and multiple sclerosis), and many of these same individuals experience neuroinflammation associated with their disorder and could have experienced a “cytokine storm” in the COVID-19 pandemic.

## Figures and Tables

**Table 1.** Baseline stimulation data

	Block 1	Block 2	Absolute Difference	%Difference	CV
Latency, ms					
TMS	17.0 ± 1.1	16.3 ± 0.8	−0.7	−4.1	8.8
CMS	7.8 ± 0.6	7.6 ± 0.5	−0.2	−2.6	3.2
Duration, ms					
TMS	54.9 ± 2.3	55.9 ± 4.1	1.0	1.8	10.9
CMS	52.3 ± 3.1	56.4 ± 2.9	4.1	7.8	5.4
Amplitude, μV					
TMS	220 ± 27	294 ± 32	74	33.6	38.8
CMS	336 ± 8	362 ± 10	26	7.7	10.6
Area, μV·ms					
TMS	1.54 ± 0.31	2.15 ± 0.35	0.6	39.6	29.9
CMS	4.32 ± 0.25	4.98 ± 0.22	0.7	15.3	10.3

Two blocks of stimulations were performed approximately 30 minutes apart before acute intermittent hypoxia to determine within-session reliability of diaphragm potentials evoked by transcranial (TMS) and cervical (CMS) magnetic stimulation. CV, coefficient of variation.

**Table 2.** Cardiovascular responses to acute intermittent hypoxia

Variable	Baseline	Episode Number				
		*3*	*6*	*9*	*12*	*15*
SpO_2_, %	98	85	85	76	82	79
HR, beats·min^−1^	89	94	90	95	96	97
SBP, mmHg	118	115	125	126	118	121
DBP, mmHg	79	78	81	80	77	80

DBP, diastolic blood pressure; HR, heart rate; SBP, systolic blood pressure; SpO_2_, oxyhemoglobin saturation.

## Data Availability

Data will be made available upon reasonable request.
